# Endothelial cells produce angiocrine factors to regulate bone and cartilage via versatile mechanisms

**DOI:** 10.7150/thno.45422

**Published:** 2020-05-01

**Authors:** Sipin Zhu, Samuel Bennett, Vincent Kuek, Chuan Xiang, Huazi Xu, Vicki Rosen, Jiake Xu

**Affiliations:** 1 Department of Orthopaedics, The Second Affiliated Hospital and Yuying Children's Hospital of Wenzhou Medical University, Wenzhou, Zhejiang, 325000, China.; 2 School of Biomedical Sciences, The University of Western Australia, Perth, WA 6009, Australia.; 3 Department of Orthopaedics, The Second Hospital of Shanxi Medical University, Taiyuan, Shanxi, 300001 China.; 4 Department of Developmental Biology, Harvard School of Dental Medicine, Boston, MA 02115, USA.

**Keywords:** angiocrine factors, angiogenic factors, bone and cartilage homeostasis, endothelial cells, angiogenesis-osteogenesis coupling

## Abstract

Blood vessels are conduits distributed throughout the body, supporting tissue growth and homeostasis by the transport of cells, oxygen and nutrients. Endothelial cells (ECs) form the linings of the blood vessels, and together with pericytes, are essential for organ development and tissue homeostasis through producing paracrine signalling molecules, called angiocrine factors. In the skeletal system, ECs - derived angiocrine factors, combined with bone cells-released angiogenic factors, orchestrate intercellular crosstalk of the bone microenvironment, and the coupling of angiogenesis-to-osteogenesis. Whilst the involvement of angiogenic factors and the blood vessels of the skeleton is relatively well established, the impact of ECs -derived angiocrine factors on bone and cartilage homeostasis is gradually emerging. In this review, we survey ECs - derived angiocrine factors, which are released by endothelial cells of the local microenvironment and by distal organs, and act specifically as regulators of skeletal growth and homeostasis. These may potentially include angiocrine factors with osteogenic property, such as Hedgehog, Notch, WNT, bone morphogenetic protein (BMP), fibroblast growth factor (FGF), insulin-like growth factor (IGF), and platelet-derived growth factor (PDGF). Understanding the versatile mechanisms by which ECs-derived angiocrine factors orchestrate bone and cartilage homeostasis, and pathogenesis, is an important step towards the development of therapeutic potential for skeletal diseases.

## Introduction

Endothelial cells (ECs)-mediated angiogenesis (the sprouting of existing vessels) plays a pivotal role in bone development, growth, and repair 1. Multiple lines of evidence indicate that bone remodelling takes place within vascularized structures, called “bone remodelling compartments” (BRCs) 2, 3. The vascular network is essential for bone formation, metabolism, and repair. Reduced bone vascularity or angiogenesis could lead to impaired bone formation, decreased bone quantity and quality, and reduced healing capacity of bone fracture 1. The reciprocal relationship of the skeleton and vascular network is regulated by complex intercellular crosstalk at the remodelling interface between bone cells (osteoblasts, osteoclasts, and osteocytes) and vascular cells (endothelial cells and pericytes) 2, 4. Bone cell-derived angiogenic factors and ECs -derived angiocrine factors are critical factors, which affect intercellular signalling and maintain homeostatic coupling of angiogenesis-osteogenesis within the bone remodelling microenvironment 5. During embryonic osteogenesis, vascularisation stimulates the replacement of the hypertrophic cartilage core by bone marrow expansion. Endochondral ossification is the process by which bones enlarge and ossify during development, occurring predominantly near the growth plate, whereby cartilage is replaced by vascularized bone tissue 6, and this process is regulated by angiogenic activity 2, 5. ECs are angiogenic progenitors of the subchondral vasculature, which provide the source for vascular expansion and secrete factors to induce late chondrocyte differentiation during endochondral ossification 7. In adult bone, the physiological processes of angiogenesis and osteogenesis are closely coupled, which is essential to maintain bone mass and homeostasis 8, 9. In pathological bone fracture, approximately 10% of human bone fractures fail to heal adequately, which may be caused by the impaired formation of blood vessels and mineralized tissue at the site of injury 10, owing in part to the disrupted intercellular signalling of angiocrine factors 11. However, the expression of angiocrine factors by ECs, and their role in skeletal homeostasis and pathogenesis remain incompletely understood.

Vascular endothelial growth factor (VEGF) signifies a potent angiogenic factor that regulates vascularized skeletal tissue throughout development, and is critical for the coupling of angiogenesis and bone formation 12-14. VEGF derived from osteoblasts appears to stimulate the osteoblastic differentiation of mesenchymal stem cells (MSCs) and bone repair 14, 15. Additional findings indicate that VEGF produced by osteoblasts also affects bone remodelling by stimulating osteoclast differentiation 14. Recently, studies have shown that osteoblasts express numerous angiogenic factors, including chemokine (C-X-C motif) ligand 9 (Cxcl9) 16, Nephronectin (NPNT) 17, EGF-like domain 6 (EGFL6) 18, EGF-like domain 7 (EGFL7) 19 and slit guidance ligand 3 (SLIT3) 20, 21; and osteoclast-like cells too express angiogenic factors, such as platelet- derived growth factor (PDGF)-BB 22 and EGFL7 19, which are involved with the mediation of angiogenesis. Notably, in the skeletal microenvironment, an array of secreted anti-angiogenic factors are also produced including chondromodulin-1 (Chm-1) 23, pigment epithelium-derived factor (PEDF) 24, and connective tissue growth factor (CTGF/CCN2) 25, that co-regulate local vascularization together with angiogenic factors, and play an important balanced role in bone and cartilage homeostasis 26-28.

Reciprocally, blood vessels produce a network of paracrine factors, called angiocrine factors, to regulate skeletal cells, such as osteoclasts, osteoblasts and chondrocytes. Several ECs-derived angiocrine factors involved with the regulation of bone have been identified, either locally within the bone microenvironment, or systemically from distal organs, such as the liver 29-32 (Table 1). Local angiocrine factors, are exemplified by canonical ligands of Notch signalling, Jagged-1 (Jag1) 33 and delta-like-4 (DLL4) 11, 34, which are produced by ECs of the bone marrow vascular niche, and are involved with the regulation of haematopoiesis and the regenerative capacity of bone tissue. Additionally, distal or systemic angiocrine factors have been reported 35, 36. For example, bone morphogenetic protein 2 (BMP2), the well-known regulator of osteoblast differentiation was found to be produced by liver sinusoidal ECs 35, 36.

Collectively, ECs are postulated to release local or systemic organ-specific angiocrine factors which orchestrate bone and cartilage homeostasis and regeneration. In this review, we will discuss ECs derived angiocrine factors, including their potential roles and mechanisms of action in the regulation of bone and cartilage homeostasis.

### Angiocrine factors of the bone microenvironment

ECs are specialized blood vessel cells present in all organ systems and are involved with the regulation of organ development and tissue homeostasis by the production of paracrine factors 37, 38. Bone marrow ECs (BMECs) regulate physiological and regenerative hematopoiesis of the bone marrow niche throughout life by the expression of angiocrine factors, including stem cell factor 1 (SCF1, also called KITL), CXCL12, and Jag1, which signal via pathways, such as NF-κB, Akt and MAPK 33, 39, 40. EC signalling within the bone marrow vascular niche is critically involved in the regulation of hematopoietic stem cell (HSC) function, both at steady state and in disease conditions 39. ECs are thought to coordinate the vital processes of bone marrow, such as osteogenesis, angiogenesis, and hematopoiesis. Recently, a subset of endoglin- expressing ECs was detected in the bone marrow, which were shown to promote the expansion of distinct subsets of hematopoietic precursor cells, ECs, and osteogenic differentiation by the abundant production of interleukin-33 (IL-33) 41.

Angiocrine factor signalling appears to provide an instructive vascular niche which is important for the modulation of the reconstitution of hematopoietic stem and progenitor cells (HSPCs), and the regulation of long-term hematopoietic stem cells (LT-HSCs) of the bone marrow 29, 31, 42. Initial studies found that angiocrine factors expressed by ECs of the vascular niche within the bone marrow microenvironment promoted the self-renewal and proliferation of LT-HSCs 30. Further, the constitutive expression of Notch ligands, such as Jag1 and jagged-2 (Jag2) was found in endothelial cells, suggesting the role of Notch signalling in the regulation of bone marrow HSPCs 30, 43.

Consistently, conditional knockout of Jag1 in ECs in mice impaired the self-renewal and led to the premature exhaustion of an adult HSC population, indicating that Jag1 expressed by the vascular niche in bone marrow regulates hematopoiesis and supports the regenerative capacity of HSCs through a Notch-dependent signalling pathway 33. Further, conditional deletion of the gene encoding Jag2 specific to ECs, indicated that Jag2 might be indispensable for regulating the hematopoietic recovery and reconstitution of HSPCs in response to myelosuppressive conditions, via Notch2-dependent signalling 43. Further, blocking DLL4, a canonical ligand of the Notch pathway family by using anti-DLL4 monoclonal antibodies appears to disturb angiogenic changes and hematopoiesis of the bone marrow 34. These data suggest that activation of Notch signalling by ECs-Jagged family proteins in HSPCs could have therapeutic potential for the enhancement of hematopoietic homeostasis after myelosuppression 43.

**Figure 1:**
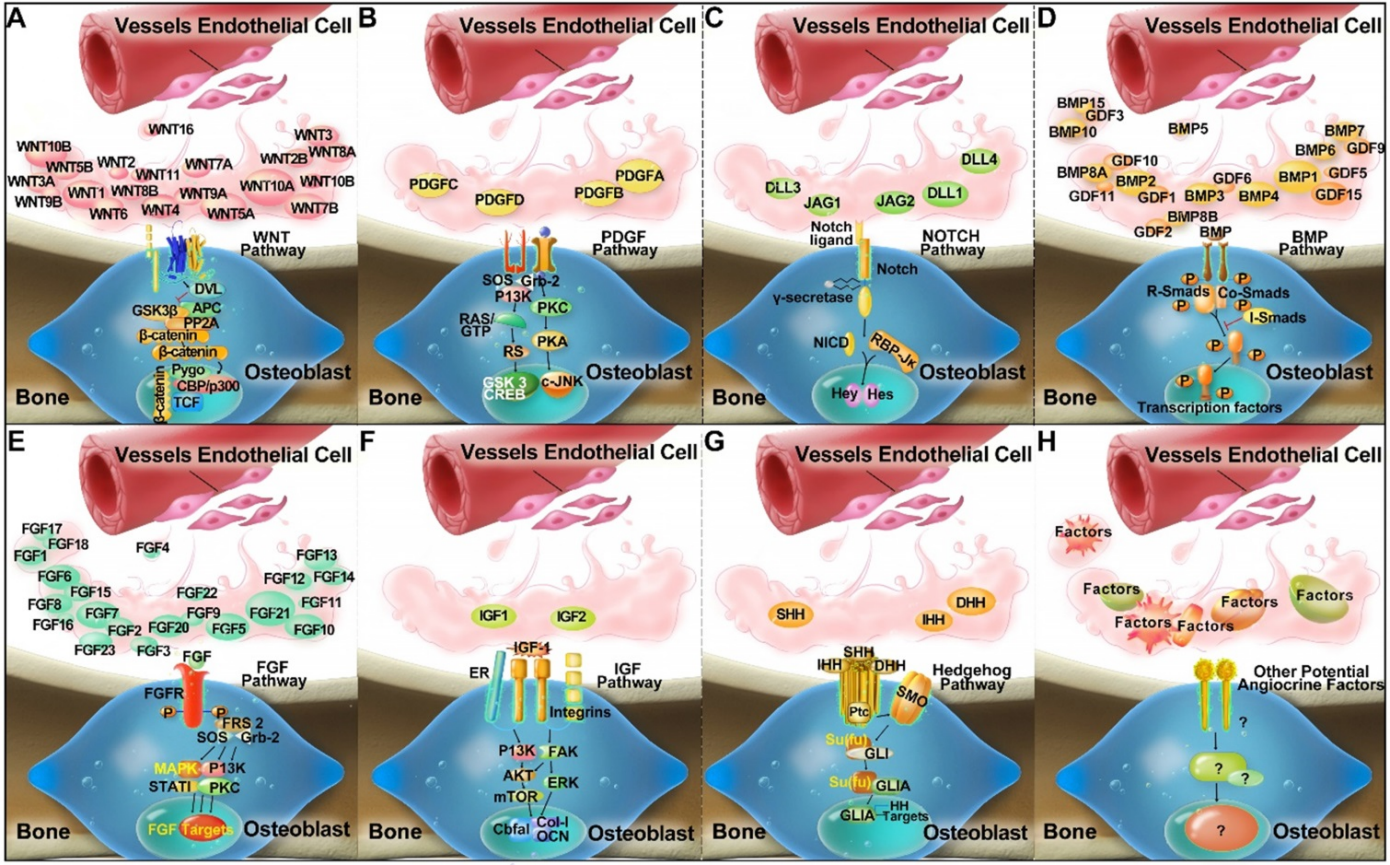
Compendium of molecules (Wnt, PDGF, Notch, BMP, FGF, IGF, and Hedgehog families) which are postulated to mediate crosstalk between ECs and osteoblastic lineage cells, and to activate putative signalling pathways via a paracrine mode of action in the bone microenvironment (A-G) by inference from experimental findings 94-102. In addition, unknown and novel angiocrine factors might be produced by ECs, which are yet to be discovered and require further research (H).

Several established receptor and ligand families appear to be important for the regulation of skeletal homeostasis and tissue regeneration, including Hedgehog, Notch, WNT, BMP, FGF, IGF, and PDGF signalling pathways 44-46. It is postulated that ECs may produce major classes of angiocrine factors at different levels, which act on their respective receptors in the osteogenic lineage cells to modulate skeletal homeostasis (Figure 1). However, it remains to be fully investigated which arrays of ECs derived angiocrine factors from other organs may have regulatory effects on skeletal growth and homeostasis.

### Potential angiocrine factors derived from organs which regulate bone via a systemic route

ECs are defined by organ-specific heterogeneity, which enables them to maintain the vital function of the local vasculature and microenvironment, such as maintenance of the blood-brain barrier, renal filtration, and hepatic clearance 35. Angiocrine factors are specific paracrine growth factors that are secreted by blood vessels, and regulate a wide range of organ development and tissue homeostasis processes, such as the maturation of retinal pigment epithelium for the establishment of the blood-retina barrier 47, thymic regeneration 48, and liver regeneration 49, 50.

An interesting question remains as to if the systemic action of angiocrine factors produced by ECs from distal organs might affect bone morphogenesis and remodelling 1, 51. For example, liver sinusoidal ECs (LSEC) are responsible for the hepatic clearance of circulation waste products, and regulate the hepatic vascular niche by producing angiocrine factors, such as BMP2 35. Genetic inactivation of angiocrine BMP2 signalling by LSECs in mice affects iron homeostasis of the liver and whole organism 36. The importance of BMP2 signalling by BMECs in bone and cartilage homeostasis, repair and regeneration is well established 52, 53. Of the BMP family, BMP2 is a prime regulator of postnatal skeletal homeostasis, and BMP2 osteogenic signalling is vital for the reparative and regenerative capacity of bone during fracture healing 52, 54. BMP2-induced osteogenesis is regulated via the activation of receptor-regulated (R-) small mothers against decapentaplegic homologs (Smads) (R-Smads), and transcription factors, such as Runx2, Osx, Dlx5, and Msx2 52, 55. Co-activation of Wnt and BMP2 signalling appears to affect osteogenic activity and the expression of Rux2 and Osx1, indicating the need for investigation of the importance of the possible distal effects of Wnt/BMP2 for skeletal homeostasis and repair 52, 56. LSECs also release inductive angiocrine factors, such as Wnt2 and hepatocyte growth factor (HGF), which stimulate hepatic regeneration 57. Given the key role of BMP2 and Wnt2 in osteogenesis, and the systemic effect of LSEC paracrine signalling of BMP2, it will be interesting to confirm the systemic effect of angiocrine factors on bone. Further investigation of the effect of inactivating BMP2 and Wnt secretion by LSECs on bone health is therefore required. Extracellular vesicles are circulating particles that contain proteins (such as BMPs, Wnts, and VEGF), and nucleic acids (such as microRNAs), and are involved in mediating intercellular communication locally and systemically 58-62. Extracellular vesicles produced by tissue-specific ECs represent a potential means of systemic delivery of angiocrine factors involved with regulating the bone microenvironment requiring further research of their therapeutic effects 63-65. For instance, recent findings indicate that extracellular vesicles from endothelial progenitor cells may have therapeutic potential for the treatment of osteoporosis 63. Further research of tissue-specific EC-extracellular vesicles and their systemic mode of action is required. Thymic endothelial cells appear to critically affect thymus repair and regeneration by BMP4 signalling, indicating the need for further research to investigate the potential for ECs-BMP4 signalling to enhance T cell immunity and skeletal health 48, 66. In responding to metabolic and pathological changes, ECs release angiocrine factors that bind to their respective receptors in a cell type dependent manner to maintain homeostasis and coordinate tissue regeneration. The expression and release of angiocrine factors by ECs is regulated by hypoxia 67-69, and mechanosensing or mechanostretching of ECs in response to blood flow 70. In addition, bone-ECs respond to endocrine signals, including parathyroid hormone (PTH), progesterone (P_4_), estrogen (E_2_), IGFs, bFGF, and PDGF 71, 72. Further investigation of the stimuli and signalling pathways affecting the expression of angiocrine factors by tissue- and organ-specific ECs, and their systemic effects is required.

### Coupling of angiogenesis and osteogenesis in bone

Although the growth of blood vessels in bone and osteogenesis appear to be coupled, the mechanisms of intercellular crosstalk regulating this relationship are largely undetermined. Specializations of the blood vessel architecture, including EC-tissue specificity, appear to take part in the coupling of angiogenesis and osteogenesis in bone 69, 73, 74.

A specific H-type capillary (CD31^hi^Emcn^hi^ ECs), was found to couple angiogenesis and osteogenesis, to regulate the metabolic and molecular microenvironment, and to sustain stem cells 69, 73. The H-type capillary (or vessel) is defined by its high expression of CD31 and Endomucin (Emcn), and is located within the metaphysis, near the growth plate, and along the periosteal and endosteal surfaces of the diaphysis, where there is an abundance of surrounding bone marrow osteoprogenitor cells, particularly platelet-derived growth factor receptor β (PDGFR-β) expressing mesenchymal cells 69, 73. H-type vessels appear to coordinate intercellular signalling between osteoblast and osteoclast lineage cells, and to produce factors for the proliferation and differentiation of osteoprogenitor cells, leading to bone formation 73. H-type vessels are an important component of the vascular hematopoietic stem cell niche, and their abundance, together with associated osteoprogenitor cells, appears to decline with age, as indicated by their crucial role in maintaining bone mass in aging mice, and their potential as an effective therapeutic target for osteoporosis and osteoarthritis treatment 69, 73, 75, 76. Further investigation of the molecular signalling by H-type vessels leading to bone formation, and the coupling of angiogenesis and osteogenesis is required 69. For example, stem cell factor (SCF) is a vital modulator of the vascular HSC niche expressed by perivascular cells, and the effect of H-type vessel signalling on SCF expression is unknown 40; and the essential role of Notch signalling by H-type vessels for the maintenance of the vascular stem cell niche remains to be elucidated 30. Factors that appear to regulate the coupling of angiogenesis and osteogenesis, and require further investigation in relation to H-type vessels, include PDGF-BB, SLIT3, hypoxia-inducible factor 1-α (HIF-1α), Notch, and VEGF 73.

Research of the molecular mechanisms mediating endothelial-osteoblast cell crosstalk demonstrates the tissue-specific angiogenesis of bone 11, 73, 75, 77. Inducible genetic disruption of Notch signalling specific to ECs in mice led to an impaired skeletal phenotype characterized by the abnormal development of blood vessels, reduced osteogenesis, shortening of the long bones, chondrocyte defects, and decreased bone mass 11. The impaired skeletal phenotype was found to involve dysregulated Notch signalling from ECs via secreted Noggin, and the delivery of Noggin was able to reverse the skeletal defects of EC-mutant mice 11. Therapeutically, halofuginone was found to attenuate the progression of osteoarthritis (OA) by mitigating articular cartilage and subchondral bone deterioration in a rodent model 74. Halofuginone appears to inhibit aberrant angiogenesis, possibly including H-type capillary formation, and uncoupled bone remodelling in the subchondral bone by reducing osteoclastic bone resorption and Smad2/3-dependent TGF-β signalling 74, 78. Halofuginone treatment is consistently associated with the reduction of expression of OA markers, such as collagen X (ColX), matrix metalloproteinase-13 (MMP13), and A disintegrin and metalloproteinase with thrombospondin motifs 5 (ADAMTS 5) 74, 79. Further, halofuginone appears to increase the expression of OA protective factors, such as lubricin, collagen II and aggrecan 74. TGF-β/Smad pathway signalling is confirmed in the pathogenesis of OA by promoting chondrocyte hypertrophy, cartilage fibrosis, mesenchymal progenitor cell differentiation to osteoblast-lineage cells, and angiogenesis within the subchondral bone by regulating downstream target genes, such as Runx2, MMP13, and ADAMTS 5 80. Therefore, halofuginone appears to disrupt several levels of OA pathogenesis affected by TGF-β signalling, and it is imperative to further investigate the molecular mechanisms of halofuginone treatment for OA in order to develop its therapeutic potential. Together these findings provide a molecular basis for the coupling of angiogenesis and osteogenesis, and highlight the vital role of bone EC-specific angiocrine factors in the bone microenvironment, thus indicating the need for further research of their potential as therapeutic targets.

### The potential role of pericytes in bone homeostasis

Pericytes, also called Rouget cells or mural cells, are located within the vascular basement membrane and closely encircle ECs in capillaries and micro- vessels 81. Pericytes are distributed throughout the organs of the body, and appear to function principally in maintaining vascular homeostasis and stability, and supporting angiogenesis in a tissue-specific manner 81-83. Studies indicate that sustained communication between pericytes, ECs, and vascular smooth muscle cells (vSMCs) maintains vascular function via Jag1 mediated Notch signalling of the Akt/mTOR pathway 83. The role of pericytes, and angiocrine factors produced by pericytes in regulating bone growth and homeostasis is largely unknown and warrants further investigation.

### The role of angiocrine factors in skeletal disease

ECs are fundamentally important in establishing the tissue-specific instructive vascular niche, whose angiocrine factors are critical for the maintenance of regional stem cell populations, and tissue repair and regeneration 30, 40. However, ECs - derived angiocrine factors may be involved in tumour growth and aggressiveness, and might be prime targets for cancer therapy and regenerative medicine 84, 85. Understanding the complex regulation of ECs - derived angiocrine factors in the bone marrow microenvironment will help to pave the way for the discovery of novel approaches to the treatment of diseases, such as leukemia. For instance, the activation of ECs by vascular endothelial growth factor-A (VEGF-A) was found to promote the proliferation of aggressive leukemic cells, and could decrease the efficacy of chemotherapeutic agents targeting leukemic cells 86; whereas inhibiting the activation of ECs by blocking VEGF-receptor 2 (VEGFR 2) signalling might increase the sensitivity of leukemic cells to chemotherapy 86. In acute myeloid leukemia (AML), the leukemia microenvironment, containing BMECs, is postulated to perpetuate refractory disease via a paracrine mechanism, and is a potential therapeutic target 87. Pazopanib, a receptor tyrosine kinase inhibitor (RTKI) of VEGFRs, PDGFRs, and cKit, was shown to be directly cytotoxic to AML cells, and to sensitize AML cells to chemotherapy by eliminating the refractory-disease effect of ECs 87. Combining RTKIs with chemotherapy therefore indicates a potential therapeutic strategy for the prevention of refractory disease and for the treatment of AML, and requires further investigation 87.

Recently, epidermal growth factor-like domain 7 (EGFL7) has been found to be involved in several types of cancers 88, 89. EGFL7 expression is involved with tissue regeneration, and loss of EGFL7 function may result in impaired vessel formation 90. Mechanistically, EGFL7 appears to bind to the extracellular matrix and act in an autocrine manner via its receptor, integrin αVβ3, to increase the motility of ECs during vessel sprouting 91. As an angiocrine factor, which is secreted by bone cells (osteoblasts and osteoclast lineages) and ECs, it may regulate bone homeostasis and cancer development 19, 90, 91, and requires further research to determine its varying roles.

In inflammation, BMECs produce cytokines, such as IL6, tumour-necrosis factor alpha (TNF-α), and interferon-gamma (IFN-γ), which induce NF-κB signalling for the proliferation and differentiation of HSPCs 39. Targeted inhibition of the NF-κB pathway in BMECs in mice following myelosuppressive injury was shown to promote hematopoietic recovery, to protect the BM micro-environment, and to limit damage of the BM vascular niche 39. Further, the transplantation of BMECs with NF-κB inactivation stimulated hematopoietic recovery and protected mice from chemotherapy-induced death by pancytopenia, indicating the potential of BMECs as a cell-based therapeutic approach for the treatment of hematological diseases 39.

In diabetic mice, bone marrow endothelial dysfunction appears to be affected by the disruption of BMEC signalling 92. Altered BMEC signalling, such as the increased activation of RhoA/Rho-associated kinase and Src/vascular endothelial cadherin pathways, and Akt inactivation, appears to dysregulate the expression of angiocrine factors by BMECs and resulted in bone marrow microangiopathy in a rodent model 92.

Additionally, circulating endothelial precursor cells, called endothelial colony-forming cells (ECFCs), represent a potential source of angiocrine factors which may improve the outcome of MSC-based regenerative applications 93. ECFCs paracrine signalling via factor PDGF-BB was found to potentiate regeneration by MSCs, and to improve MSC transplantation 93. MSC engraftment was regulated by signalling from the interaction between PDGF-BB and its receptor, PDGFR-β, and was enhanced by the co-transplantation of ECFCs 93. Further research is needed to investigate the possible application of ECFC-enhanced tissue regeneration in a disease-specific manner.

Future research of angiocrine signalling by ECs, such as BMECs, ECFCs, and the identification of novel factors and their mechanisms of action under pathological conditions, will help us to develop new therapeutic targets and regenerative medical treatments for diseases, including leukemia, osteoporosis, inflammation, and diabetes 32.

## Conclusions

**Figure 2:**
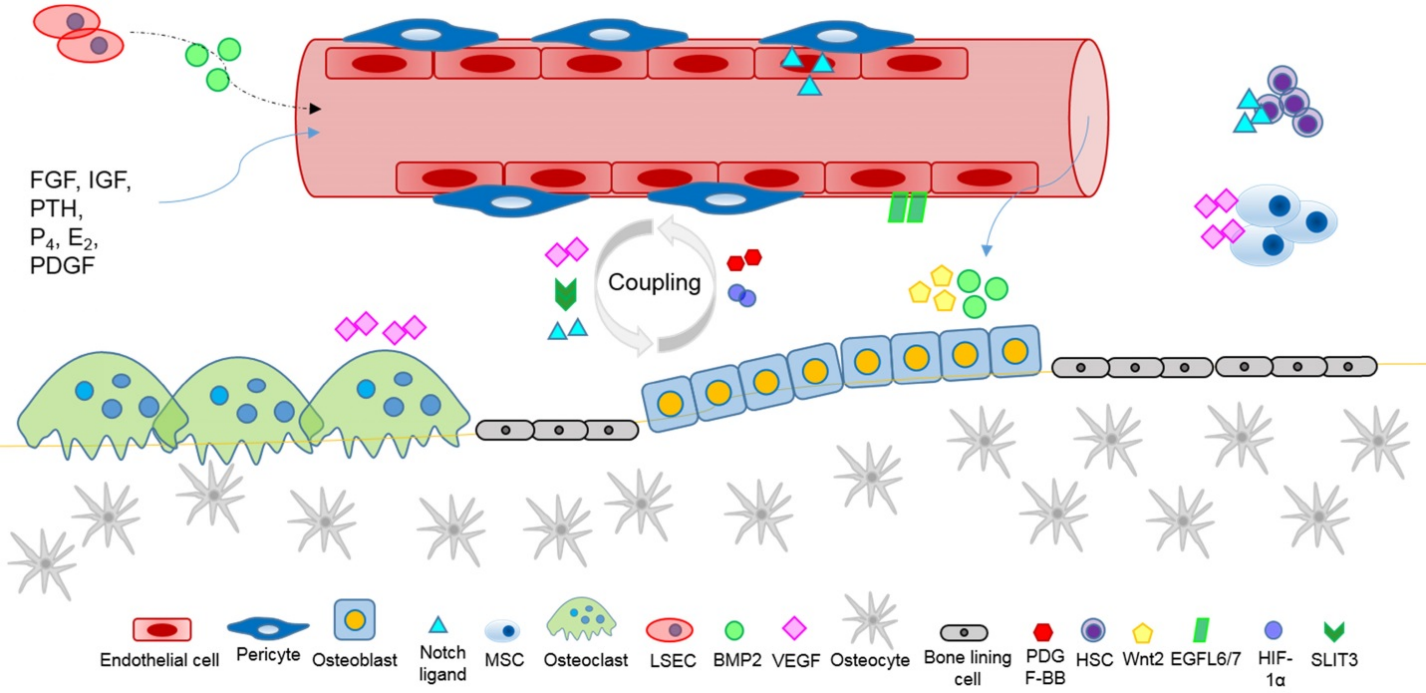
Angiocrine factors mediate intercellular communication within the bone microenvironment affecting the regulation of skeletal homeostasis and repair, involving the coupling of angiogenesis and osteogenesis, and systemic paracrine signals.

Bone growth, development, homeostasis and repair, require the exchange of oxygen, nutrients, and metabolites with the dense vascular system that surrounds and permeates skeletal tissue, and the anoxic bone microenvironment. Reciprocal crosstalk between skeletal cells (osteoblasts, osteoclasts, osteocytes), blood vessel cells (endothelial cells and pericytes), and bone marrow progenitor cells coordinate the physiological processes of bone growth and remodelling, vascular development, and the vital coupling of angiogenesis and osteogenesis. Angiogenic factors, produced by skeletal cells, affect local vascular ECs and angiogenesis (Figure 2). Conversely, angiocrine factors, secreted by vascular cells locally and systemically, appear to mediate skeletal development, homeostasis, and the recruitment of stem cells during wound healing. Several major classes of ligands, including Hedgehog, Notch, WNT, BMP, FGF, IGF, and PDGF are likely to mediate intercellular crosstalk for the regulation of skeletal homeostasis, and to affect the pathogenesis of skeletal diseases. Angiocrine factor signals of the instructive vascular niche, are involved with the regulation of bone marrow hematopoiesis, enhance the regenerative capacity of HSCs, and help to maintain a healthy bone marrow microenvironment. Future research elucidating the complex crosstalk of angiogenic and angiocrine signals, will help to shed new light on ECs metabolism, organ-specific vascular networks and tissue homeostasis, and advance our knowledge and capability to target vasculature disorders in skeleton, such as impaired fracture healing, osteonecrosis and cancer.

## Figures and Tables

**Table 1:** Examples of angiocrine factors produced locally in the bone microenvironment and by distal organs.

Angiocrine factors	Produced by endothelial cells	Local and/or distal effects	References
	**In bone microenvironment**	**Local effect**	
Delta-like 4	Sinusoidal endothelial cells	Hematopoietic cell modulation	34
Kit-ligand	Sinusoidal endothelial cells	Hematopoietic stem and progenitor cells (HSPCs)	40
SDF1	Sinusoidal endothelial cells	Hematopoietic stem and progenitor cells (HSPCs)	50
Jagged-1	Sinusoidal endothelial cells	Self-renewal and regenerative capacity of hematopoietic stem cell	33
Jagged-2	Sinusoidal endothelial cells	Homeostasis of haematopoietic stem and progenitor cells	43
Interleukin-33	Sinusoidal endothelial cells	Expansion of hematopoietic precursor cells, and osteogenic differentiation	41
Angiocrine factors	Sinusoidal endothelial cells	Long-term hematopoietic stem cells	29, 30
Angiocrine factors	Sinusoidal endothelial cells	Hematopoietic stem and progenitor cells (HSPCs)	31
Noggin, BMPs, Jagged-1	Type-H and L vessels	Osteogenic differentiation	11
			
	**In distal organs**	**Local and possible distal effect**	
BMP2	Liver sinusoidal endothelial cells	Iron homeostasis of the liver, possible osteogenic differentiation	35, 36
Wnt2	Liver sinusoidal endothelial cells	Hepatic regeneration, possible osteogenic differentiation	57
BMP4	Thymic endothelial cells	Thymic regeneration, possible osteogenic differentiation	48

## Abbreviations

BMEC: bone marrow endothelial cell
BMP: bone morphogenetic protein
BRC: bone remodelling compartment
CXCL: C-X-C motif ligand
EC: endothelial cell
EGF: epidermal growth factor
E_2_: estrogen
FGF: fibroblast growth factor
HSC: hematopoietic stem cell
HSPC: hematopoietic stem and progenitor cell
IGF: insulin-like growth factor
LSEC: liver sinusoidal endothelial cell
MAPK: mitogen-activated protein kinase
MSC: mesenchymal stem cell
NF-κB: nuclear factor kappa-light-chain-enhancer of activated B cells
PDGF: platelet-derived growth factor
P_4_: progesterone
TGF: transforming growth factor
VEGF: Vascular endothelial growth factor
